# Microbiological Health Risk Assessment of Water Conservation Strategies: A Case Study in Amsterdam

**DOI:** 10.3390/ijerph18052595

**Published:** 2021-03-05

**Authors:** Agung Kusumawardhana, Ljiljana Zlatanovic, Arne Bosch, Jan Peter van der Hoek

**Affiliations:** 1 Department of Water Management, Faculty of Civil Engineering and Geosciences, Delft University of Technology, P.O. Box 5, 2600 AA Delft, The Netherlands; AgungKusumawardhana@student.tudelft.nl (A.K.); L.Zlatanovic-1@tudelft.nl (L.Z.); 2 Amsterdam Institute for Advanced Metropolitan Solutions, Kattenburgerstraat 5, 1018 JA Amsterdam, The Netherlands; 3 Water Supply Company Noord-Holland PWN, Rijksweg 501, 1991 AS Velserbroek, The Netherlands; 4 Waternet, P.O. Box 94370, 1090 GJ Amsterdam, The Netherlands; arne.bosch@waternet.nl

**Keywords:** QMRA, greywater reuse, rainwater harvesting, drinking water, toilet flushing, showering, cross connection, human health risk

## Abstract

The aim of this study was to assess the health risks that may arise from the implementation of greywater reuse and rainwater harvesting for household use, especially for toilet flushing. In addition, the risk of cross connections between these systems and the drinking water system was considered. Quantitative microbial risk assessment (QMRA) is a method that uses mathematical modelling to estimate the risk of infection when exposure to pathogens happens and was used in this study to assess the health risks. The results showed that using rainwater without prior treatment for toilet flushing poses an annual infection risk from *L. pneumophila* at 0.64 per-person-per-year (pppy) which exceeds the Dutch standard of 10^−4^ pppy. The use of untreated greywater showed a risk that is below the standard. However, treatment is recommended due to the ability of *P. aeruginosa* to grow in the reuse system. Moreover, showering and drinking with cross-connected water has a high annual infection risk that exceeds the standard due to contact with *Staphylococcus aureus* and *E. coli* O157:H7. Several measures can be implemented to mitigate the risks such as treating the greywater and rainwater with a minimum of 5-log removal, closing the toilet lid while flushing, good design of greywater and rainwater collection systems, and rigorous plumbing installation procedures.

## 1. Introduction

The increase in global population leads to an increasing demand for nutrients and energy. However, the raw materials to produce the needed nutrients are becoming scarce and expensive [1]. Moreover, urban water demand is also expected to rise, stressing the available drinking water sources that are already limited [2]. On top of that, climate change is expected to alter precipitation patterns regarding its frequency, duration, strength, and spatial range. Not only do long periods of drought lead to shortages of water supply, they can also make water source quality worse due to less dilution [3,4,5]. Thus, a way is needed to secure nutrients and water supply in the future.

Thankfully, wastewater contains recoverable resources such as energy, water, nutrients, cellulose fibers, biopolymers, bioplastics, and protein [6]. However, recovering resources from municipal wastewater remains a challenge due to its dilute nature [7]. One way to make resource recovery more effective is by concentrating the wastewater by using less drinking water. The options are through using water saving appliances and recycling greywater [8], which may contribute to alleviating the stress of available drinking water sources.

In addition to greywater reuse, rainwater harvesting is another method that has been proposed to reduce drinking water demand [9,10], although it will not result in a more concentrated wastewater [8]. Both greywater and rainwater offer an additional source for water usage in households that does not require drinking water quality such as flushing toilets. The use of these sources for toilet flushing has been found to reduce drinking water demand by around 20% to 30% [9,11]. Furthermore, drinking water saving can also be obtained by using greywater and rainwater for washing machines [12].

Nevertheless, greywater and rainwater should be used with caution since undesired pathogens may be present in greywater and rainwater. These pathogens can cause gastrointestinal diseases such as diarrhea and pulmonary disease such as pneumonia [13,14]. Humans can be exposed to pathogens through different exposure routes: respiratory, digestion, and dermal contact routes, depending on the form of water usages. However, the probability of pathogens to infect humans depend on the dose of exposure and the response of the human body to a particular pathogen [15]. Aside from pathogens, various organic micropollutants are also present in greywater and rainwater. In greywater, at least 278 organic micropollutants have been identified [16,17]. However, this study does not cover health risk assessment of the chemicals.

In this study microbial health risk assessment will be covered, following the quantitative microbial risk assessment (QMRA) method that estimates the risk of infection from pathogens using mathematical models [18].

Previous studies have discussed the health risk of using greywater or rainwater for various usages. However, most of them only focused on one or two particular pathogens in one study, most notably *Legionella pneumophila* [19,20,21,22]. In addition, most of them also did not indicate the level of pathogens removal that is needed to get the risk at a safe level and what measures can be applied to mitigate the risks. Only one research was found that has specifically studied the pathogen reduction target [23].

The case study area in this research is Prinseneiland, Amsterdam, where a project was conducted to study the changes that would happen to current urban water infrastructures with the implementation of various water conservation scenarios to enable resource recovery from wastewater. The water conservation scenarios include implementation of water saving appliances such as 1 L flush toilets and water saving showers, and reuse of greywater and rainwater harvesting for toilet flushing and washing machines. Research has been carried out into hydraulics and wastewater composition [8]. However, the health risks of the implementation of water conservation scenarios have not been assessed yet.

Thus, this study aimed at assessing the health risks of the implementation of water conservation strategies using quantitative microbial risk assessment (QMRA). Furthermore, if the infection risk of a pathogen exceeded the permissible level, the level of removal that is needed to reduce the risk was also calculated. To make the assessment more comprehensive, measures that can be done to mitigate the risks were also discussed.

## 2. Materials and Methods

### 2.1. QMRA Method

For microbial health risk assessment, the quantitative microbial risk assessment (QMRA) method was followed that estimates the risk of infection from pathogens using mathematical models. The first step in QMRA is to identify and select target pathogens for the exposure scenario of interest. After that, the dose of each target pathogen is estimated for each exposure scenario. Additionally, then the probability of infection is estimated using dose–response models corresponding to each target pathogen. Lastly, the estimated infection risk is compared to a benchmark value [18].

### 2.2. Hazard Identification

#### 2.2.1. Description of Scenarios

Two water conservation scenarios were considered in this study. The first is reuse of greywater from bathroom and washing machine, and the second is rainwater harvesting. The greywater and rainwater would be used for toilet flushing. In addition, the reuse of greywater and use of rainwater necessitate the installation of a dual plumbing system that introduces a risk of cross connection between the greywater reuse system or rainwater system and the clean drinking water system. The risk assessment for cross-connections was done for the case of using greywater or rainwater instead of drinking water for showering, and for the case of using greywater or rainwater instead of drinking water for water consumption.

#### 2.2.2. Target Pathogens in Greywater

Greywater includes wastewater from laundries, bathtubs, showers, bathroom sinks, and kitchen sinks [24]. Skin and mucous tissue pathogens such as *Staphylococcus aureus* and *Pseudomonas aeruginosa* can be found in greywater from bathing and laundries [25]. Aside from that, *Salmonella* spp. and *Shigella* spp. may also be found in greywater originating from food handling processes [20]. Moreover, pathogenic *E. coli* O157:H7 and enteric viruses have also been found in greywater [26]. In this study, greywater is collected from bathroom and washing machine, thus the relevant pathogens in this case are *Staphylococcus aureus, Pseudomonas aeruginosa,* and *Escherichia coli* O157:H7. Even though viruses may also present in greywater, we could not find enough data regarding their concentration in greywater, thus viruses are not included in this study.

*L. pneumophila* in greywater is also not covered in this study due to its limited data availability. Even though several studies have found *L. pneumophila* in the drinking water distribution system which ultimately can end up in the greywater system, the data are difficult to compare due to differences in the water systems, climate, and environmental factors of each study locations [27]. Moreover, the data on amoeba, which can be attributed to the proliferation of *L. pneumophila* in the greywater system is still lacking [28]. Although Blanky [28] have quantified *L. pneumophila* in greywater, their study location is in Israel which has different climatic and environmental factors compared to this study’s case area.

#### 2.2.3. Target Pathogens in Rainwater

Rainwater harvesting involves collecting rainwater from a catchment, storage, and the use of the collected water. Microbial contaminants can be introduced from the air, the surface of the catchment, the conveyance system, and in the storage [29]. Various pathogens have been identified in harvested rainwater such as *E. coli*, *Salmonella* spp., *Giardia lamblia, Legionella* spp., *Campylobacter jejunii, Aeromonas* spp., *Pseudomonas* spp., *Mycobacterium avium complex* (MAC), and *Naegleria fowleri* [13,21,30,31]. Even though viruses may also present in rainwater, we could not find enough data regarding their concentration in rainwater, thus viruses are not included in this study.

At least two authors have quantified the concentration of pathogens in roof harvested rainwater in Australia. It was found that *Legionella spp.,* MAC, and *Pseudomonas aeruginosa* are the most abundant pathogens [13,32]. These pathogens are also a concern for their infection route through inhalation [33,34,35]. Due to the availability of concentration data and the possibility of infection through inhalation, the pathogens that were selected as the target pathogens in rainwater are *Legionella spp.,* MAC, and *Pseudomonas aeruginosa.* Moreover, to take into account the possibility of infection through drinking water, *Escherichia coli* O157:H7 was also considered as target pathogen.

### 2.3. Exposure Assessment

#### 2.3.1. Concentration of Pathogens

The pathogen concentration data for this study that are shown in Table 1 were compiled from various literatures. The concentration range of pathogens in Colony Forming Units (CFU) was needed to calculate the risk of infection using dose–response models. However, the concentration data of MAC and *L. pneumophila* are only available in gene copies unit. It was assumed that one gene copy is equivalent to one viable cell since the PCR primer sets for MAC and *L. pneumophila* targeted a single copy gene [32]. Moreover, data on the concentration of *E. coli* O157:H7 in greywater and harvested rainwater is lacking. Therefore, concentration of *E. coli* was used to estimate the concentration of *E. coli* O157:H7. The ratio between the concentration of *E. coli* O157:H7 and the concentration of *E. coli* was assumed to be 0.027 [36].

After that, the concentration distribution of pathogens was constructed using lognormal distribution as recommended by WHO [37]. Once the distribution was constructed, the mean and standard deviation of the lognormal were calculated. The mean and standard deviation were used as the input for risk characterisation calculations. Construction of lognormal distribution of pathogen concentrations was carried out using Matlab.

#### 2.3.2. Exposure Routes

**Figure 1:**
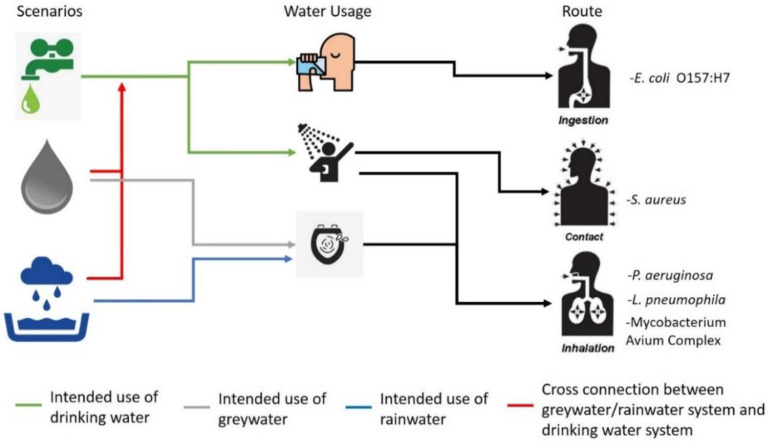
Illustration of exposure routes.

The main exposure pathway in this study is through toilet flushing. During toilet flushing, aerosolization of water happens and inhalation of aerosolized water is possible [46]. Moreover, errors in the plumbing installation may be present, where cross connection between the greywater/rainwater system and the drinking water system may happen. Cross connection cases have been reported in the Netherlands where residents became ill due to cross connections between the household water system and drinking water system [47]. In case of cross connections, contaminated drinking water will enter the human body through ingestion of drinking water. Furthermore, showering with contaminated drinking water due to cross connections can also expose humans to pathogens through inhalation and dermal contact route. The route of exposure by which pathogens enter the human body in this study is illustrated in Figure 1.

#### 2.3.3. Exposure Dose

The exposure dose is the number of pathogens that enters the human body. Although the formula for each exposure route is different, the basic is the same: concentration of pathogens times the volume of water that is ingested, inhaled, or adsorbed. The values for each parameter in the formulas were derived from literature and are presented in Table 2.

- Ingestion route

The dose of pathogens that is ingested is described by Equation (1) [23]. The exposure dose (*D*) is a function of pathogen concentration in water (*C*) multiplied by the volume of ingested water (*V_in_*) and the numbers of events per day (*N*).

(1) $$D=\;C\;x\;V_{in}\;x\;N$$

where:

*D* = daily dose unit (CFU)
*C* = concentration of pathogens in water (CFU/L)
*V_in_* = volume of ingested water per exposure event (L)
*N* = number of events per day

b. Inhalation route

For the dose of pathogens that is inhaled, other factors are also accounted for as can be seen in Equation (2) [34]. A partitioning coefficient was used to estimate the concentration of pathogens that is aerosolized. The volume of aerosols that is inhaled was calculated using inhalation rate and duration. Aerosols are generated in different droplet size and only certain size can enter the respiratory system [14]. This is represented with the fraction of respirable aerosols and retention rate [34].

(2) $$D=\;C\;x\;PC\;x\;IR\;x\;T\;x\;F_{RA}\;x\;RR\;x\;N$$

where:

*D* = daily dose (CFU)
*C* = concentration of pathogens in water (CFU/L)
*PC* = partitioning coefficient (L/m^3^)
*IR* = inhalation rate (m^3^/minutes)
*T* = duration of exposure event (minutes)
*F_RA_* = fraction of respirable aerosol
*RR* = retention rate
*N* = number of events per day

c. Dermal contact route

As for dermal contact route, it has been found that short contact times (0.1–30 min) had little to no influence on pathogen transfer [48]. Adsorption of pathogens to the skin (represented in Equation (3)) is influenced by the concentration of pathogens in water and the thickness of water on the skin after contact with water [48,49]. It is assumed that drying the body with a towel will leave no water on the skin after showering.

(3) $$D=\;C\;x\;\left(10^{-3.38}+h\right)\;x\;BSA\;x\;N$$

where:

*D* = daily dose (CFU)
*C* = concentration of pathogens in water (CFU/mL)
*h* = thickness of water on skin after showering (cm)
*BSA* = Body surface area (cm^2^)
*N* = number of events per day
Calculating for log removal

Calculation of dose with log removal follows Equation (4):

(4) $$DLR=D\;x\;10^{-LR}$$

where:

*DLR* = daily dose after certain log removal (CFU)
*D* = daily dose (CFU)
*LR* = Log removal

### 2.4. Dose–Response

The dose–response assessment is done to determine the connection between the exposure level to pathogens and the probability of adverse effects. A dose–response model is a mathematical function that takes a value of dose and generates the probability of infection, illness, or other adverse effects [51]. The mathematical model that is used to model dose–response relationships varies according to the target pathogens. The two commonly used models are the exponential model and the beta-poisson model. The dose–response models that were used in this study were selected from previous peer reviewed journal articles and are shown in Table 3. An exponential model was used for *Staphylococcus aureus, Pseudomonas aeruginosa, Legionella pneumophila*, and Mycobacterium Avium Complex, whereas a beta-poisson model was used for *E. coli* O157:H7. Whether an exponential or a beta-poisson model is used for certain pathogens is because certain models fit better to the dose–response data of the pathogen compared to other models [33,52,53,54,55].

- Exponential model

The exponential model is the simplest dose–response model. In this model, it is assumed that each organism has the same constant probability of survival, represented by a variable k. The value of k will be different for every pathogen. The general formula is shown in Equation (5).

(5) $$P_{Infection}=1-e^{-k.d}$$

where:

*P_infection_* = probability of infection
*k* = probability of survival and reaching the host of the pathogen
*d* = dose (CFU)

b. Beta-poisson model

The exponential model has a limitation because it ignores the variation of infectivity between pathogens and variation of human responses. This variation is accounted for in the beta-poisson model by allowing the *k* value to be governed by a probability distribution. The general formula is shown in Equation (6).

(6) $$P_{Infection}=1-{\left(1+\frac{d\;\left(2^{1/\alpha }-1\right)}{N_{50}}\right)}^{-\alpha }$$

where:

*P_infection_* = probability of infection
*α* = variable α
*N*_50_ = the dose level at which 50% of the population is expected to be affected
*d* = dose (CFU)

### 2.5. Risk Characterisation

Annual risk of infection for each pathogen and exposure route scenario was calculated using Equation (7) [56]. Monte Carlo simulations with 10,000 iterations were done using MATLAB software (MathWorks, Natick, USA) to calculate the annual probability of infection and the results are shown using boxplots. The simulation code was based on risk characterization flowcharts developed by Shi et al. [36]. The calculated probability of infection was then compared to the infection risk limit of 10^−4^ as described in the Dutch drinking water regulation [57] and the water is deemed unsafe if the infection risk limit is exceeded [37].

(7) $$P_{Inf,ann}=1-\overset{f}{\underset{1}{\prod }}\left(1-P_{Inf,daily}\right)$$

where:

*P_inf,ann_* = annual probability of infection
*P_inf,daily_* = daily probability of infection
*f* = frequency

## 3. Results and Discussion

The results of the risk characterisation for the use of greywater and rainwater for toilet flushing and for the case that cross connection with drinking water takes place are presented in this section. In short, the use of greywater and rainwater without prior treatment poses a significant risk of infection from different pathogens depending on the route of exposure. To mitigate the risk, several measures that can be employed are discussed further in this section.

### 3.1. Risk Characterisation

#### 3.1.1. Toilet Flushing

**Figure 2:**
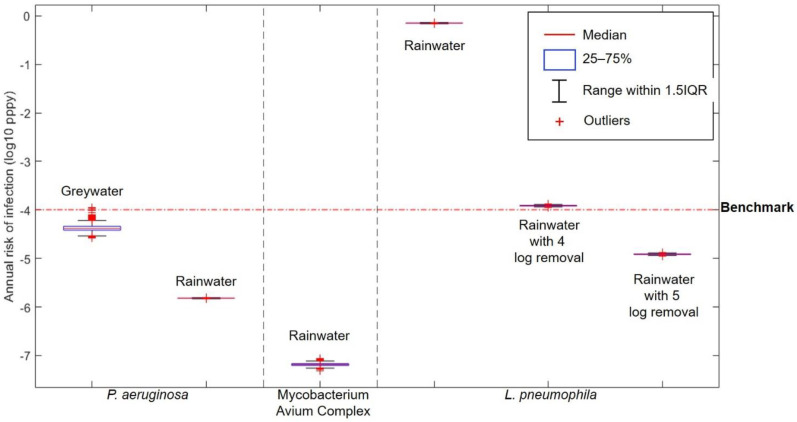
Annual infection risk from toilet flushing.

The result of risk characterisation for the toilet flushing exposure route is shown in Figure 2.

The boxplots as descriptive statistics clearly show the annual risks from using untreated and treated greywater and rainwater compared to the infection benchmark of 10^−4^ per-person-per-year (pppy). The results show that the 95th percentile of the annual risk from using untreated greywater contaminated with *P. aeruginosa* for toilet flushing is 0.54 × 10^−4^ pppy which is slightly below the infection benchmark of 10^−4^ pppy. Although the annual infection risk from the use of untreated greywater for toilet flushing is below the benchmark, a previous study has shown that *P. aeruginosa* can regrow in the reuse system even if the greywater has been treated [58]. If *P. aeruginosa* can grow, then the concentration in the toilet reservoir will be higher compared to the concentration in untreated greywater, thus increasing the risk of infection.

Annual infection risks of *P. aeruginosa* and MAC in untreated harvested rainwater are below the benchmark at around 10^−6^ pppy and 10^−7^ pppy, respectively. In contrast, the annual risk of infection from *L. pneumophila* is way above the benchmark at 0.71 pppy. To get the annual infection risk of *L pneumophila* below the benchmark, treatment with 5-log removal is needed. Compared to *L. pneumophila*, both *P. aeruginosa* and MAC were present in lower concentrations in rainwater (see Table 1). Moreover, the infectivity of both these pathogens, represented with k values in the dose–response relationship are way lower than *L. pneumophila*.

However, it should be noted that the concentration of *L. pneumophila* was measured in gene copies unit. Overestimation of the infection risk is highly probable by assuming that one gene copy is equivalent with one viable cell [21]. The use of the partitioning coefficient to calculate the dose of exposure may also have contributed to the overestimation of infection risk in this study. Compared to using aerosol size distribution, partitioning coefficients tend to result in higher infection risks [59].

Furthermore, the use of the partitioning coefficient in this study did not take into account the variability of toilet flush volume. It was found that the amount of aerosols generated from toilet flushing is affected by the flush volume and flush energy. Flush energy itself cannot be measured directly, and it is used to describe the degree of water agitation during flushing. Higher number of aerosols are generated from higher flushing energy [46]. Thus, the use of dual flush toilets will generate different amount of aerosols depending on which volume is used and the use of water saving toilets can reduce the risk of infection.

The median concentration of *L. pneumophila* in this study is 1.7 × 10^3^ gc/100 mL which was assumed to be equal to 1700 CFU/100 mL. A previous study found that the median critical concentration of *L. pneumophila* to cause 10^−4^ pppy annual risk of infection in conventional toilets and water efficient toilets is 103 CFU/100 mL and 168 CFU/100 mL, respectively [59]. The concentration of *L. pneumophila* in this study is 10 times higher than the critical concentration calculated by Hamilton [59], thus an annual infection risk exceeding the benchmark can be expected. However, the annual risk of infection in this study is almost 4-log higher than the benchmark, indicating an overestimation of risk.

Although we did not assess the risk of *L. pneumophila* in greywater due to lack of data, Blanky [19] assessed the use of raw greywater contaminated with *L. pneumophila* for toilet flushing in Israel. Concentrations of *L. pneumophila* in that study ranged from 7.1 × 10^2^ CFU/100 mL to 2.9 × 10^4^ CFU/100 mL. Benchmark of 10^−5^ illness-cases-per-person-per-year (ippy) tolerable annual disease risk was used in that study and it was found that the annual disease risk for *L. pneumophila* in raw greywater used for toilet flushing exceeded the benchmark at 1.3 × 10^−4^ ippy.

#### 3.1.2. Cross Connection—Showering

**Figure 3:**
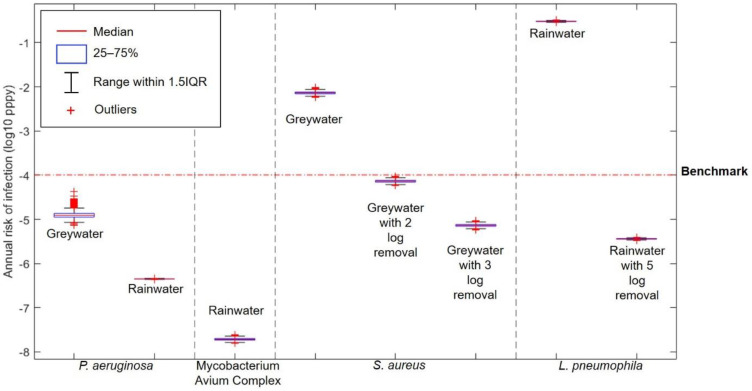
Annual infection risk from showering with cross connected water.

The result of the risk characterisation for showering with cross connected water is shown in Figure 3.

For *P. aeruginosa* and Mycobacterium Avium Complex, the results for the risk of showering with greywater or rainwater (Figure 3) show that the risk of infection by inhalation route is lower than from toilet flushing (Figure 2). Showering with untreated greywater contaminated with *P. aeruginosa* resulted in a risk of infection of 0.60 × 10^−4^ pppy at 95th percentile, below the 10^−4^ pppy benchmark. Moreover, as is the case with toilet flushing, showering with water connected to the rainwater harvesting system poses a high risk of infection to *L. pneumophila* with an annual risk way above the benchmark. Treatment of rainwater with 5-log removal of *L. pneumophila* resulted in annual infection risk of 0.36 × 10^−5^ pppy, which is below the benchmark.

Comparison of annual infection risk of *L. pneumophila* between showering and toilet flushing has been done in a previous study. Hamilton [59] found that the annual risk of infection of showering is higher than toilet flushing. It should be noted that Hamilton [59] compared a conventional shower and a water efficient toilet in their study, using aerosol size distribution to calculate dose of exposure. As has been discussed before, the use of water efficient toilets generates less aerosols and can reduce the risk of infection.

Compared to a previous study by Dean & Mitchell [34], the annual risk of infection from *P. aeruginosa* in this study is higher. Although the concentration of *P. aeruginosa* in this study is about 7-log lower than in Dean & Mitchell’s study, the annual infection risk is only 1-log lower. Dean & Mitchell conducted a reverse QMRA by first specifying the target infection risk to know the maximum permissible range of pathogen concentration and found that a median concentration of *P. aeruginosa* at 6.04 × 10^10^ CFU/100 mL resulted in annual infection risk of 10^−4^ pppy. The median concentration of *P. aeruginosa* in this study is 3.89 × 10^3^ CFU/100 mL and resulted in an annual infection risk of 10^−5^ pppy which is below the benchmark, and the water can be considered safe to use.

In contrast to the inhalation route, the dermal contact exposure route by *S. aureus* resulted in a high annual infection risk that exceeds the benchmark at 0.73 × 10^−2^ pppy. However, there is still an uncertainty in this result as the adsorption rate of *S. aureus* to skin is still not available. The *E. coli* adsorption rate found by Pitol [49] was used instead as the adsorption rate for *S. aureus*. The uncertainty comes from the differences between the two bacteria, as *E. coli* is a Gram-negative bacteria, and *S. aureus* is a Gram-positive bacteria. It has been observed that there is a difference of transfer efficiencies between Gram-positive and Gram-negative bacteria to skin [49]. Therefore, future study on the adsorption rate of *S. aureus* to skin is needed to get a more certain result.

#### 3.1.3. Cross Connection—Drinking Water Consumption

**Figure 4:**
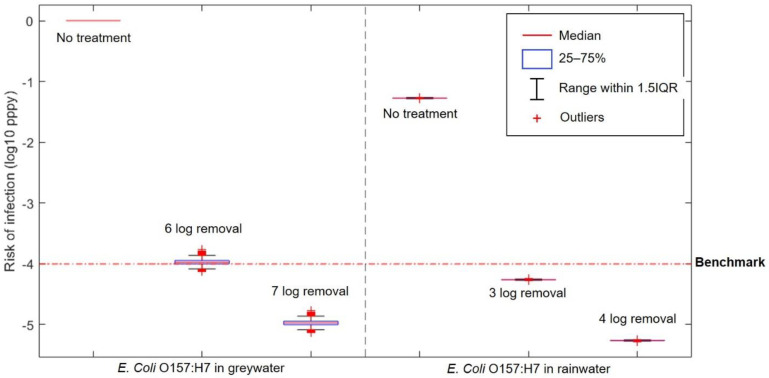
Annual infection risk of drinking from cross connected water.

The result of the risk characterisation for drinking contaminated water is shown in Figure 4. Out of all target pathogens that are considered in this study, only *E. coli* O157:H7 can infect human through ingestion route.

Both untreated greywater and rainwater pose a great risk of infection when cross connection to drinking water happens. Only one day of exposure from drinking untreated greywater and rainwater already has an infection risk of 0.08 per-person-per-day and 14 × 10^−4^ per-person-per-day, respectively (daily infection risk not shown in graph) which is way above the benchmark of 10^−4^ pppy. Treatment with 6 and 7-log removal of *E. coli* from greywater were simulated, resulting in an annual infection risk below the benchmark for 7-log removal. As for rainwater, 3-log removal is needed to get the annual infection risk below the benchmark.

Compared to toilet flushing, and showering with contaminated water, drinking contaminated water presents the greatest risk for infection. Consuming this water just for one day already generates an infection risk above 10^−4^. To avoid infection risks exceeding the benchmark, the requirement of 7-log removal for greywater will be costly, especially on household scale. Therefore, cross connection between greywater reuse or rainwater harvesting systems and drinking water systems must be avoided.

### 3.2. Uncertainty of Pathogens Concentrations

There are some uncertainties regarding the concentration of pathogens in greywater and rainwater since the data was taken from secondary sources. Among households, activities that produce greywater vary. Washing machine load may differ between households, and bathing practices may also differ. These may result in varying levels of pathogens in greywater. Furthermore, it has been found that the concentrations of indicator *E. coli* in greywater were highly variable [26].

The microbial quality of harvested rainwater varies with seasonal and climatic condition. In this regard, temperature plays an important role in the growth of microbes, and higher concentrations of opportunistic pathogens were found in rainwater storage tanks in areas with higher temperatures [60].

In this study, we did not model these uncertainties and variabilities. However, future studies may consider modelling these uncertainties and variabilities to get a more comprehensive picture of microbial risks from the use of greywater and rainwater.

Aside from temperature, antecedent dry periods also play a role in the accumulation of animal feces deposits on the roof or dry deposition of particles that can carry microorganism, and thus may increase the concentration of pathogens in runoff. [61]. It has also been observed that higher rainfall intensity correlates with increasing concentration of pathogens [62]. In addition, roofing material also affects the microbial community of harvested rainwater. It was found that harvested rainwater from metal and clay tiles roof contained the least pathogens compared to other materials [63,64].

The method for the measurement of pathogenic *E. coli* is complex and direct measurement is rarely done [34]. In this study, pathogenic to total *E. coli* ratio of 0.027 was used based on previous assumptions in a study by Shi [36]. They came up with this number based on a study by O’Toole [26] that detected virulence gene markers among *E. coli* isolates. It was found that around 3% of samples were positive for pathogenic *E. coli*. However, no concentration value has been reported [26].

### 3.3. Risk Mitigation Measures

#### 3.3.1. Greywater Reuse

The collection of greywater must be made in such a way that no blackwater can enter the greywater reuse system. In time of maintenance of the system, a bypass must be provided to convey greywater into the blackwater or wastewater sewerage system. Storage of raw greywater should be avoided or minimized to prevent the growth of microbes in the greywater [65]. Furthermore, the storage tank of treated greywater must be covered and protected from sunlight, and periodically cleaned [66].

#### 3.3.2. Rainwater Harvesting

Strainers can be installed in the rain gutter to retain large organic material and dirt [67]. After that, installation of a first flush device can be done to reduce the pathogens in the rainwater that will be collected in the rainwater harvesting tank. First flush devices work by diverting and flushing off the first runoff from roof. The volume of first flush depends on rainfall intensity and dry days prior to rainfall event. Optimizing the first flush device based on these two factors is important to optimally divert pollutants from rainwater storage and in general, 0.1 to 3.8 mm of rainfall needs to be flushed to get a good quality rainwater [67,68]. Nevertheless, the capability of first flush devices is limited as pathogens were still found in the rainwater tank after first flushing [69].

Sizing of the rainwater tank based on the calculation of supply and demand can be done to avoid undersizing or oversizing the rainwater tank. In the rainwater tank, suspended materials and pathogens that may be attached to the suspended materials can settle, and sludge may form on the bottom of the rainwater tank. Proper positioning of the outflow pipe must be considered in order to avoid taking and disturbing the sediments. Resuspension of sediments can also be mitigated by designing a proper inlet that can avoid turbulence. Periodic desludging and tank cleaning should also be done as bacteria can grow in the sludge. Another way to minimize microbial growth is keeping the rainwater storage covered and positioned in a place where low temperature can be maintained [67].

#### 3.3.3. Treatment Options

Treatment is needed to ensure that the greywater or harvested rainwater is safe to be used for non-potable usage such as toilet flushing. The most common treatment system for greywater is coarse filtration followed by disinfection [70]. Other treatment options for greywater are biological treatment using rotating biological contactors (RBC), fluidized bed bioreactors, or membrane bioreactors (MBR) [71,72,73]. As for harvested rainwater treatment systems, slow sand filtration, solar pasteurisation, and disinfection are commonly used [70].

Household slow sand filtration has been found to be able to remove 3-log of *E. coli* [74]. In another study, slow sand filtration has been found to be able to remove more than 2-log of *L. pneumophila* [75]. Although sand filtration and membrane treatment can reject pathogens, regrowth of pathogens has been observed [58]. Furthermore, some pathogens may also not be completely removed by membrane treatment and sand filtration. Therefore, disinfection is needed to ensure the necessary removal requirement [76].

UV_254_ disinfection with doses of 3 mJ/cm^2^ have been found to inactivate 3-log of *L. pneumophila* [77]. For inactivation of *P. aeruginosa*, slightly higher doses of 5 mJ/cm^2^ were needed to achieve 3-log inactivation [78]. As long as no recirculation system is involved, UV doses of 20 mJ/cm^2^ are sufficient to reduce 4-log of bacteria. However, if a recirculation system is involved such as using treated greywater for washing machine and treating the washwater again, resistance of bacteria to disinfection can happen and higher UV doses might be needed [79]. Aside from UV_254_ disinfection, chemical disinfection using chlorine has also been effective to inactivate pathogens and make the greywater or rainwater safe for non-potable use [28,71].

All these treatment options focus on the inactivation and removal of pathogens, used in the QMRA applied in this study. The presence of viruses and organic micropollutants in greywater and harvested rainwater were out of the scope of this study. Hence, the efficacy of these treatment options for risks related to these contaminants is not considered and requires future research.

It should be noted that operation of treatment plants on a household level by the homeowners poses a risk of failure due to inadequate maintenance by the homeowners. However, the risk can be managed if there are adequate management strategies of the treatment plants [80]. An example of the strategy is the one implemented by New South Wales, where installation of greywater treatment systems is permissible after accreditation has been done. There is also a penalty system if the homeowners do not properly maintain the system [81]. Another example is in Singapore, where greywater reuse systems are not allowed on individual household scale but allowed for a larger scale. There, the maintenance can only be done by an authorized service contractor [66]. Nevertheless, further study is needed to formulate the management strategies if this system is to be implemented because management strategies should be site specific [80].

#### 3.3.4. Cross Connection

Dual plumbing systems have been implemented in various locations such as Florida, California, Fukuoka, Tokyo, Sydney, New South Wales, and Queensland. This kind of system must be managed well due to the risk of cross connections [82]. Several cross-connection events have been reported not only in the Netherlands [47], but also in other locations. In Australia, cross-connection events have occurred in Rouse Hill, Sydney Olympic Park, Pimpama-Coomera, and a place in Melbourne. Cross-connections have also occurred in Nokia, Finland, resulting in 6500 illnesses [82]. Even though multiple cross-connection events have been reported, this matter has not been studied much.

Preventing cross connections requires a solid standard procedure for reuse system installations and rigorous plumbing inspection. Colour coding or clearly marking the pipes and plumbing equipment according to their water source can be a way to prevent erroneous installation. Furthermore, the installation of drinking water pipes and recycled water pipes should be separated at some distance. When laid horizontally, the drinking water pipes should always be positioned above the recycled water pipes [66]. Other management practices that can be implemented include: (i) limiting installation and modification of the system only to licensed individuals; (ii) applying pressure differential to ensure that if a cross connection happens, water flows from the drinking water to the reuse water; (iii) education to explain the necessity of preventing cross connections [83].

Early detection of cross connections is important to manage the risk of cross connections. During commissioning of the system, tracer tests as dye testing can be used to detect cross connections. If coloured water comes out from the drinking water system, there is a cross connection [66]. Recently, real time detection of cross connections between reclaimed water systems and potable water systems using machine learning methods (pearson correlation coefficient—supporting vector machine) has been developed and found to be effective and reliable [84].

#### 3.3.5. Toilet Flushing

Preventing contact with aerosols during flushing can be done by closing the lid of the water closet [85]. Another measure that can be done is installing water saving toilets as they can reduce the generation of aerosols and subsequently reduce the aerosolization of pathogens. Aside from flush volume, flushing energy also affects aerosol generation. It was found that high efficiency toilets generate less aerosols compared to pressure-assisted high efficiency toilets with the same flush volume [46].

## 4. Conclusions

Reuse of greywater and use of rainwater may be attractive strategies to enhance resource recovery opportunities from wastewater and to offer an additional source for drinking water production, but are also characterized by human health risks. In this study the use of greywater and rainwater for toilet flushing was considered. In addition, cross connections were considered that lead to the use of greywater or rainwater for showering or drinking water consumption. The use of water conservation strategies introduces microbiological health risks above the benchmark value of 10^−4^ pppy. The following detailed conclusions were made based on the risk evaluation of these systems:

1. Inhalation of aerosols from toilet flushing is the main exposure route to pathogens, with *Legionella pneumophila* as the major pathogen that causes high level of infection risk.
2. The risk of infection from *P. aeruginosa* in untreated greywater for toilet flushing is below the benchmark of 10^−4^ pppy. However, treatment of greywater is recommended due to the ability of *P. aeruginosa* to regrow in reuse systems, even after disinfection. Harvested rainwater can also be used for toilet flushing after sufficient treatment through 5-log removal of *L. pneumophila* is done.
3. Cross connections between drinking water and greywater/harvested rainwater systems pose a high daily risk of infection from *E. coli* O157:H7 through drinking and a high annual risk of infection from *Staphylococcus aureus* and *Legionella pneumophila* through showering.
4. To mitigate the microbial risks, several measures can be implemented. The simplest measure is keeping the toilet lid closed during and after flushing. Collection and storage of greywater and rainwater should be managed well to prevent excessive growth of pathogens in the storage system. Cross-connections can be avoided through rigorous plumbing installation and test procedures.

This study only covered several bacterial pathogens due to availability of data. To get a more comprehensive assessment on the safety of greywater or harvested rainwater, more pathogens which are not yet covered in this study such as viruses need to be assessed in future studies. To be able to cover more pathogens, more data is needed such as the concentration of *L. pneumophila* in greywater and viruses from several locations. Moreover, further research is also needed to address the limitation of this study regarding the adsorption of pathogens through skin, which is important to assess dermal infection of pathogens such as *S. aureus*. Finally, a study into the health risks due to chemical compounds in rainwater and greywater should complement this study.

## Figures and Tables

**Table 1:** Pathogen concentrations.

Pathogens	Source	Min	Max	Unit	Lognormal Mean	Lognormal Std	Reference
*S. aureus*	Greywater	120	1.58 × 10^4^	CFU/100 mL	7.23	1.0	[38,39]
*P. aeruginosa*	Greywater	94	1.57 × 10^5^	CFU/100 mL	8.25	1.51	[38,40]
*P. aeruginosa*	Rainwater	200	900	CFU/100 mL	6.05	0.18	[41,42]
MAC	Rainwater	22	6.80 × 10^4^	gc/100 mL	7.11	0.95	[21]
*L. pneumophila*	Rainwater	300	9.80 × 10^3^	gc/100 mL	7.45	0.41	[21]
*E. coli* O157:H7 *	Greywater	540	2.10 × 10^5^	CFU/100 mL	9.29	1.23	[43,44]
*E. coli* O157:H7 *	Rainwater	5	25	CFU/100 mL	2.41	0.19	[41,45]

* Based on a ratio of 0.027 between the concentration of *E. coli* O157:H7 and the concentration of *E. coli* [36].

**Table 2:** Exposure parameter values.

Variable	Value	Unit	Reference
Partitioning coefficient of aerosol for toilet flushing	2.3 × 10^−5^	L/m^3^	[19]
Partitioning coefficient of aerosol for showering	1.07 × 10^−5^	L/m^3^	[34]
Inhalation rate of aerosol	0.013	m^3^/minutes	[34]
Respirable fraction of aerosols	U(0.963, 0.997)	-	[34]
Retention rate of aerosol from toilet flushing	U(0.38, 0.58)	-	[21]
Retention rate of aerosol from showering	U(0.34, 0.44)	-	[34]
Duration of toilet flushing	U(1, 5)	minutes	[21]
Duration of showering	U(7.8, 17)	minutes	[34]
Volume of ingested water for drinking	2	L	[23]
Flush frequency	5	/day	[8]
Thickness of water after showering	0 *	cm	
Body surface area	N(16,168.8, 6277.81)	cm^2^	[50]

Notes: N = normal distribution; U = uniform distribution; * = use of towel.

**Table 3:** Pathogen dose–responses.

Target Pathogens	Model	Parameters	Values	Reference
*Staphylococcus aureus*	exponential	*k*	8.5 × 10^−8^	[52]
*Pseudomonas aeruginosa*	exponential	*k*	3.22 × 10^−7^	[53]
*Legionella pneumophila*	exponential	*k*	0.06	[54]
MAC	exponential	*k*	3.12 × 10^−9^	[33]
*E. coli* O157:H7	Beta-poisson	*α* *N*_50_	1.55 × 10^−1^ 2.11 × 10^6^	[55]

## Data Availability

The data presented in this study are available on request from the corresponding author.
